# Air quality prediction model based on mRMR–RF feature selection and ISSA–LSTM

**DOI:** 10.1038/s41598-023-39838-4

**Published:** 2023-08-07

**Authors:** Huiyong Wu, Tongtong Yang, Hongkun Li, Ziwei Zhou

**Affiliations:** College of Science, Shenyang University of Chemical Technology, Shenyang, Liaoning China

**Keywords:** Data processing, Machine learning

## Abstract

Severe air pollution poses a significant threat to public safety and human health. Predicting future air quality conditions is crucial for implementing pollution control measures and guiding residents' activity choices. However, traditional single-module machine learning models suffer from long training times and low prediction accuracy. To improve the accuracy of air quality forecasting, this paper proposes a ISSA–LSTM model-based approach for predicting the air quality index (AQI). The model consists of three main components: random forest (RF) and mRMR, improved sparrow search algorithm (ISSA), and long short-term memory network (LSTM). Firstly, RF–mRMR is used to select the influential variables affecting AQI, thereby enhancing the model's performance. Next, ISSA algorithm is employed to optimize the hyperparameters of LSTM, further improving the model’s performance. Finally, LSTM model is utilized to predict AQI concentrations. Through comparative experiments, it is demonstrated that the ISSA–LSTM model outperforms other models in terms of RMSE and R^2^, exhibiting higher prediction accuracy. The model's predictive performance is validated across different time steps, demonstrating minimal prediction errors. Therefore, the ISSA–LSTM model is a viable and effective approach for accurately predicting AQI.

## Introduction

As economic growth progresses, there is a sharp increase in energy demand, which brings along potential dangers of unsustainable resource extraction in developing countries. The adverse consequences of urbanization, such as deforestation, escalating waste generation, and air pollution, are becoming increasingly evident. These environmental issues, particularly the deteriorating air quality causing problems like smog, acid rain, and ozone depletion, pose significant threats to the pursuit of sustainable development. Faced with these challenges, one of the key tasks is to accurately forecast air quality. By leveraging advanced technologies like predictive modeling, machine learning, and data analysis, reliable models for air quality prediction can be developed. These predictive models can provide valuable insights for policymakers and environmental agencies, aiding in the formulation and implementation of effective measures to mitigate air pollution and promote sustainable development.

The air quality status is commonly represented using the AQI. Traditional methods for predicting AQI include time series models^1^, linear regression models^2^, and the widely used Autoregressive Integrated Moving Average (ARIMA) model^3^. Slini et al.^4^ achieved favorable predictive results by using the ARIMA model to forecast the maximum ozone concentration in Athens, Greece. Their research demonstrated the effectiveness of the ARIMA model in predicting ozone levels. In recent years, with the advancement of statistical theories and the versatility of machine learning, its applications have expanded across various fields, from healthcare and finance to transportation and marketing. Leveraging its powerful data processing and analysis capabilities, machine learning has revolutionized the decision-making process, improved efficiency, and unlocked new innovative opportunities. Consequently, researchers have increasingly adopted machine learning methods such as support vector regression (SVR)^5^ and random forest^6^. Dai et al.^7^ proposed a typical hybrid VAR-XGBoost model to estimate the spatio-temporal distribution of O_3_.Yu et al.^8^ employed the RF to predict AQI in urban areas. Song et al.^9^ used an ARIMA-SVM hybrid model to forecast PM_2.5_ concentrations in Shenyang. Samia et al.^10^demonstrated the effectiveness of a composite model combining Artificial Neural Networks (ANN) and ARIMA. By harnessing the capabilities of ANN and ARIMA, the composite model showcased its ability to capture nonlinearity and time dependencies, thereby improving the accuracy of air quality forecasts. Gao et al.^11^ proposed an enhanced model called MFO-SVM, which utilized the Firefly Optimization Algorithm to enhance Support Vector Machines (SVM). The optimized model demonstrated effective prediction of the AQI, significantly improving accuracy compared to single-model approaches. Yan et al.^12^ utilized Backpropagation (BP) neural networks to train and learn the measured pollutant concentrations in Xi'an, ultimately obtaining satisfactory predictive results. Jiang et al.^13^ proposed a BP based on sample self-organizing clustering, utilizing the clustering features of self-organizing competitive neural networks to predict air quality. The model achieved improved prediction accuracy. While the final predictive results showed significant improvement in accuracy, BP neural network models still have some drawbacks, such as slow convergence speed.

LSTM has been widely used in AQI due to its ability to effectively utilize long-term temporal information and adequately consider the relationship between nonlinear factors and time series data. Bai et al.^14^ employed LSTM to predict PM_2.5_ levels in the air. Zhang et al.^15^ introduced a novel fusion of VMD-BiLSTM within a hybrid deep learning framework. This innovative approach aimed to predict PM_2.5_ concentrations at monitoring stations in Beijing using time-series data. Belavadi et al.^16^ utilized a combined model integrating LSTM, RNN, and wireless sensor networks for air quality prediction. Gilik Aysenur et al.^17^ developed a composite forecasting model called CNN-LSTM and applied it to analyze data on various pollutants in cities such as Barcelona, Kolkata, and Istanbul. The results demonstrated that the composite model outperformed traditional LSTM models, exhibiting higher prediction accuracy and stability.

One of the key steps in setting up and training a neural network model is defining the model's hyperparameters. Choosing inappropriate parameters can lead to issues of overfitting or underfitting, thereby hindering the desired performance. To enhance prediction accuracy, swarm intelligence algorithms are employed to optimize the model's hyperparameters. Swarm intelligence optimization algorithms possess global search capabilities, robustness, and generalization abilities, enabling them to handle multi-objective optimization problems. Leveraging the advantages of swarm intelligence algorithms such as PSO^18^ or WOA^19^ significantly improves the process of searching and fine-tuning hyperparameters. These algorithms facilitate exploration of a broader range of parameter combinations and identify optimal configurations, thereby improving model performance and prediction accuracy. The simplicity and ease of implementation of the SSA algorithm make it an attractive choice for optimization problems, and This approach has found extensive applications in diverse domains, encompassing engineering, finance, and data analysis. Jiang et al.^20^ used SSA to optimize an autoregressive recurrent network (DeepAR) to build an SSA-DeepAR model to predict atmospheric PM_2.5_ concentrations.

The rapid process of urbanization and environmental pollution has raised concerns about air quality. Numerous studies have been devoted to developing air quality prediction models to anticipate potential air quality issues in advance. These studies have explored various environmental variables, such as temperature, humidity, wind speed, wind direction, and atmospheric pressure, which influence air quality. However, existing research still faces challenges in selecting input variables and improving prediction accuracy. To address these challenges, this study proposes an innovative air quality prediction model that combines RF-mRMR and LSTM. The RF-mRMR is employed to select important variables, reducing the number of input variables and improving model efficiency. Subsequently, an ISSA is used to optimize the hyperparameters of the LSTM, enhancing the predictive performance of the model. The constructed model, named ISSA–LSTM, utilizes the selected important variables to forecast future air quality indices. To ascertain the efficiency and versatility of the ISSA–LSTM, it is applied and validated in two cities as case studies. The performance evaluation of the model encompasses different time scales, including 2-step and 4-step ahead air quality index predictions. Through validation experiments, the accuracy and stability of the model are assessed, providing decision support for urban environmental management.

The paper is structured as follows: "Introduction" section introduces the research background and discusses the methodologies used by previous scholars in the field. "Materials and methods" section presents the research methodology, providing a detailed explanation of the components involved. In "Experiments and analysis" section, the data sources are described, and a performance analysis is conducted on the ISSA–LSTM composite model, comparing it with other models like LSTM and CNN et al. Finally, "Conclusions" section presents the concluding remarks drawn from the study.

## Materials and methods

### Building ISSA–LSTM model

**Figure 1:**
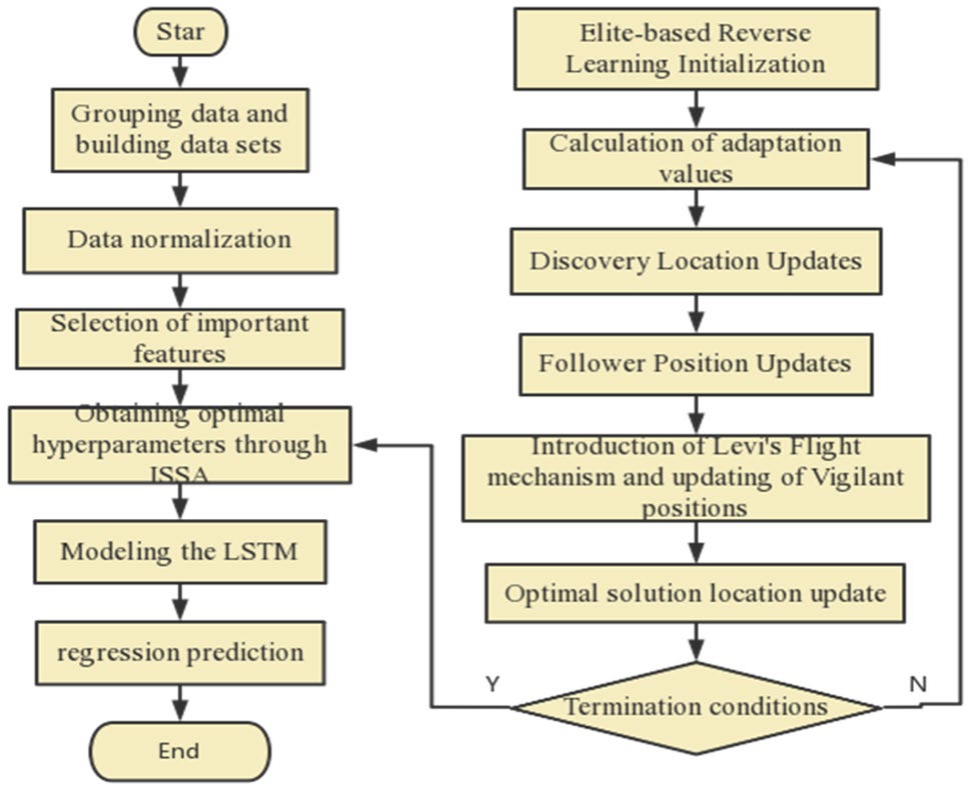
Flowchart of the composite model.

The paper proposes a method based on mRMR-RF feature selection and ISSA–LSTM model. In the feature selection stage, the advantages of both filter-based and wrapper-based algorithms are combined, providing good generalization performance, computational efficiency, and low computational cost, while improving the model performance. Firstly, the filter-based algorithm mRMR is used to calculate the mutual information between features and between features and class variables, sorting the features accordingly. Then, the wrapper-based algorithm RF is employed to calculate the out-of-bag data error values and further rank the feature importance. By testing the impact of different feature numbers on the model accuracy, the optimal number of features, *k*, is determined. Finally, the results of the two feature selection methods are integrated to select the top *k* features, constructing an optimal feature subset. Furthermore, the elite backward learning is applied to compare the current solution with the backward solution in the ISSA–LSTM algorithm, selecting superior individuals for the next iteration and accelerating the convergence speed. By introducing the Golden Sine mechanism to improve the explorer's search strategy, the model can comprehensively explore high-quality solution spaces. Additionally, the Lévy flight strategy is employed to introduce perturbation and conduct local search near the optimal position, aiding in escaping local optima and enhancing the local search capability of the sparrow optimization algorithm. The ISSA–LSTM prediction model is ultimately created by employing ISSA to optimize LSTM's hyperparameters. Figure 1 presents the flowchart of the proposed composite model.

### LSTM

LSTM is an improvement over traditional recurrent neural networks (RNNs) that addresses the issues of vanishing and exploding gradients. It introduces the concepts of cell state and hidden state, where the cell state changes slowly while the hidden state changes significantly. This design enables LSTM to better handle long-term dependencies.

To control the content of the cell state, LSTM incorporates gate mechanisms, including the input gate, forget gate, and output gate. These gate mechanisms allow LSTM to selectively add or remove information from the cell state, thereby controlling the flow of information. The functions of each gate mechanism are as follows:

- The forget gate determines which information from the previous time step's output should be discarded or forgotten. It consists primarily of a Sigmoid structure that produces output values ranging between 0 and 1. After undergoing a linear transformation, the input is selectively forgotten using the Sigmoid function. This mechanism enables LSTM to retain or discard past memories as needed, better capturing long-term dependencies in time series data.
- The input gate is composed of two parts: an information update part and a part that determines which parts need to be updated. The first part involves a Sigmoid layer that determines the specific values to be updated. The second part involves creating a candidate vector computed using the current input sequence information. This vector is used to update the cell state and improve data representation. By selectively updating relevant information, LSTM efficiently captures extended relationships in data and addresses challenges related to diminishing and amplifying gradients.
- The output gate utilizes a Sigmoid layer to determine the relevant output-related information. The neuron state is updated by multiplying it with the hyperbolic tangent of the output from the Sigmoid layer. Subsequently, the output gate multiplies the updated neuron state with the results from the prior time step to obtain the desired output. The output gate generates a new output based on the current input and state, which becomes the input for the next sequence step.

**Figure 2:**
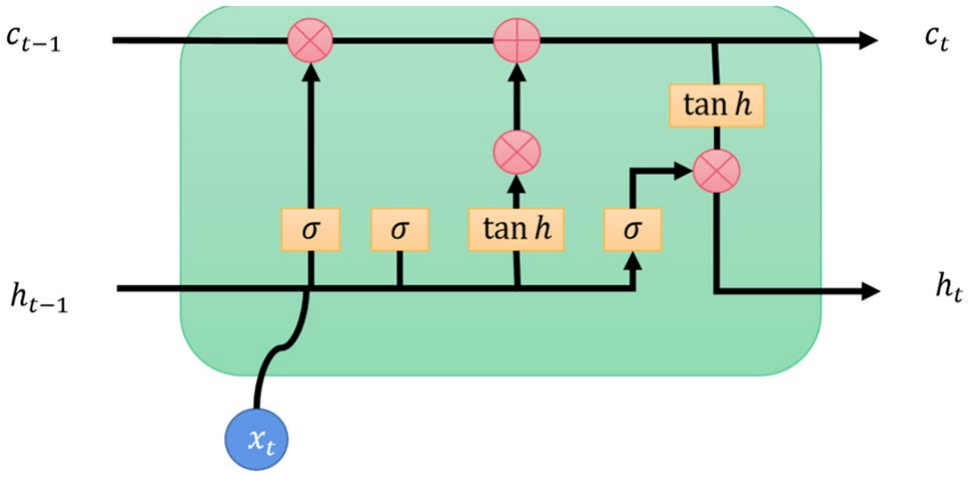
LSTM schematic.

The core of LSTM lies in the cell state, which is visualized in the model architecture diagram. Figure 2 clearly illustrates the components of LSTM and the flow of information. Through this design, LSTM effectively captures long-term dependencies when processing sequential data, leading to significant performance improvements in various natural language processing and time series prediction tasks.

1 $$\begin{array}{l}\begin{array}{l}{f}_{t}=\sigma \left({W}_{xf}{x}_{t}+{W}_{hf}{h}_{t-1}+{b}_{f}\right)\end{array}\end{array}$$

2 $$\begin{array}{l}{i}_{t}=\sigma \left({W}_{xi}{x}_{t}+{W}_{hi}{h}_{t-1}+{b}_{i}\right)\end{array}$$

3 $$\begin{array}{l}{c}_{t}{\prime}=\tanh\left({W}_{xc}{x}_{t}+{W}_{hc}{h}_{t-1}+{b}_{c}\right)\end{array}$$

4 $$\begin{array}{l}{c}_{t}={f}_{t}\odot {c}_{t-1}+{i}_{t}\odot {c}_{t}{\prime}\end{array}$$

5 $$\begin{array}{l}{o}_{t}=\sigma \left({W}_{xo}{x}_{t}+{W}_{ho}{h}_{t-1}+{W}_{co}{c}_{t}+{b}_{c}\right)\end{array}$$

6 $$\begin{array}{l}{h}_{t}={o}_{t}\cdot tanh\left({c}_{t}\right)\end{array}$$

In the above six formulas, Where $\odot$ is the vector inner product, $i$ and $f$ are the input gate and the forgetting gate; $c$ and $o$ are cell state and output gate; $W$ refers to the corresponding weight coefficients and $b$ represents the bias values; The activation function $\sigma$ refers to the sigmoid function, while the hyperbolic tangent function is denoted by tanh; ${h}_{t}$ is the output at time $t$.

### Feature selection based on mRMR-RF

#### RF

RF is an aggregate classifier consisting of multiple decision trees, each with independent parameters ${\theta }_{k}$. The decision trees in the Random Forest determine the optimal classification result through voting. To compute the relative importance of features, perform feature ranking, and selection, Random Forest utilizes the Out-of-Bag (OOB) method. This method calculates the importance values for each feature, facilitating feature ranking. Feature selection is a process of ranking all features based on different rules to determine their importance. The Random Forest algorithm evaluates the impact of each feature on model accuracy by introducing noise and observing the decrease in accuracy. This determines the feature's importance. The basic steps for feature selection in Random Forest include computing feature importance values, sorting, and filtering the features. Valuable feature information can be obtained to support model prediction and decision-making processes. The specific steps for feature selection are as follows:

- *Step 1*: Compute the error values of each decision tree using k sets of OOB data, denoted as $Er{r}_{OOB}1$, $Er{r}_{OOB}2$, $\cdots$, $Er{r}_{OOB}k$;
- *Step 2*: Randomly permute the $i$ feature of the $k$ sets of OOB data while keeping the other features unchanged, and recalculate the error values;
- *Step 3*: Calculate the importance of each feature ${X}_{i}$;
  7 $$\begin{array}{l}IM{PORTANCE}_{i}=\frac{1}{k}\sum_{i=1}^{k}\left({S}_{k}-{S}_{k,i}\right)\end{array}$$
- *Step 4*: Sort the features based on their importance and select the top m features according to the determined optimal number of features, m.

In formula (7), ${S}_{k}$ represents the OOB error rate prior to the modifications of the feature, ${S}_{k,i}$ represents the OOB error rate subsequent to the modifications of the feature.

#### mRMR

The maximum relevance minimum redundancy (mRMR) algorithm is a type of feature selection method that operates as a filter by striking a balance between relevance and redundancy. It employs mutual information as the criterion to measure the redundancy between features and the relevance between features and the class variable. By maximizing the correlation between features and the class variable while minimizing the redundancy among features, the mRMR algorithm aims to identify the optimal feature set. Following the principle of maximum relevance, this algorithm selects the features that exhibit the highest correlation with the model. As higher correlation indicates a stronger ability of the model to solve problems, the calculation of maximum relevance involves a mathematical formula used to quantify the correlation between features and the class variable. Through the utilization of this algorithm, we are able to effectively choose the most informative features, thereby enhancing the performance and accuracy of the model. The formula for calculating maximum relevance is expressed as:

8 $$\begin{array}{l}maxD\left(S,c\right),D=\frac{1}{\left|S\right|}\sum_{{x}_{i\in s}}I\left({x}_{i},c\right) \end{array}$$

In the aforementioned formula: ${x}_{i}$ represents the ith feature, $c=\left\{{c}_{1},{c}_{2},\cdots ,{c}_{L}\right\}$ represents the class variables, $L$ denotes the total number of classes, and $S$ denotes the feature subset.

The higher the correlation between features, the greater the redundancy. To mitigate redundancy, each feature should possess representativeness. By applying the principle of minimum redundancy and its corresponding formula, we can assess the degree of redundancy among features. The objective of this principle is to minimize redundancy. The formula for calculating minimum redundancy is as follows:

9 $$\begin{array}{l}\mathrm{min}R\left(S\right), R=\frac{1}{{\left|S\right|}^{2}}\sum_{{x}_{i},{x}_{j\in S}}I\left({x}_{i};{x}_{j}\right) \end{array}$$

### SSA

SSA was first developed by Xue and Shen in 2020, which primarily replicates how sparrows forage in nature. There are three different types of individuals in the entire sparrow population: discoverers, followers, and vigilant individuals. Discoverers are in a position to provide the entire community with the location of food and the location of predators since they have enough energy reserves. Followers in the population will follow discoverers to search for food. When encountering danger, the vigilant individual will issue a warning to other companions. During the entire foraging process, the optimal individual in the group will have priority access to food. Compared with followers, discoverers can search a larger search space and find more food. Therefore, the entire population is led by the discoverers' movement. In simulation experiments, virtual sparrows are utilized for food searching purposes. A group consisting of only sparrows be represented in a particular format:

10 $$\begin{array}{l}X=\left[\begin{array}{cccc}{x}_{1}^{1}& {x}_{1}^{2}& \cdots & {x}_{1}^{d}\\ {x}_{2}^{1}& {x}_{2}^{2}& \cdots & {x}_{2}^{d}\\ \cdots & \cdots & \cdots & \cdots \\ {x}_{n}^{1}& {x}_{n}^{2}& \cdots & {x}_{n}^{d}\end{array}\right]\end{array}$$

where $d$ stands the dimensionality of the parameter and $n$ denotes the count of sparrows. The mathematical expression for the fitness of each sparrow is as follows:

11 $$\begin{array}{l}{F}_{X}=\left[\begin{array}{l}f\left(\left[\begin{array}{cccc}{x}_{\mathrm{1,1}}& {x}_{\mathrm{1,2}}& \cdots & {x}_{1,d}\end{array}\right]\right)\\ f\left(\left[\begin{array}{cccc}{x}_{\mathrm{2,1}}& {x}_{\mathrm{2,2}}& \cdots & {x}_{2,d}\end{array}\right]\right)\\ \vdots \\ \vdots \\ f\left(\left[\begin{array}{cccc}{x}_{n,1}& {x}_{n,2}& \cdots & {x}_{n,d}\end{array}\right]\right)\end{array}\right]\end{array}$$

In the above formula, $f$ represents the fitness value.

Discoverers in a sparrow population exhibit strong adaptability, enabling them to not only seize food resources promptly but also effectively lead the entire sparrow population in their foraging trajectory. Consequently, discoverers have a broader search range compared to followers. The update equation for the position of a discoverer can be represented as follows, If ${R}_{2}$ is less than $S$, it indicates the absence of natural predators, allowing the discoverer to perform an extensive search. However, if ${R}_{2}$ is greater than or equal to $S$, it signifies that certain sparrows have already detected the presence of a natural enemy and issued a warning to their companions. In such circumstances, all members of the population must promptly evacuate the area and relocate to other safe locations in search of food.

12 $$ \begin{array}{*{20}l} {X_{i,j}^{t + 1} = \left\{ {\begin{array}{*{20}l} {X_{i,j}^{t} exp\left( {\frac{ - i}{{\alpha t_{max} }}} \right)} & {\left( {R_{2} < S} \right)} \\ {X_{i,j}^{t} + QL} & {\left( {R_{2} \ge S} \right)} \\ \end{array} } \right.} \\ \end{array} $$

where $t$ and ${t}_{max}$ represent the current iteration and maximum Iterations, respectively. ${X}_{i,j}^{t}$ represents the position of the tth individual in the j-dimensional space at the tth iteration, The random number $\alpha \in \left(\mathrm{0,1}\right]$, $L$ is a matrix of $1\times d$ and each element is 1; $d$ is the dimension, ${R}_{2}\left({R}_{2}\in \left[\mathrm{0,1}\right]\right)$ is the warning value; $S\left(S\in \left[\mathrm{0.5,1}\right]\right)$ is the safety value, $Q$ is random value with a normal distribution integer.

Followers closely monitor the discoverers during the food search process. When followers observe that the discoverers have located more favorable food sources, they promptly abandon their current positions and engage in competition for the food. If successful, followers acquire the food initially discovered by the discoverers. However, if unsuccessful, they must relocate to alternative areas to resume their search for food. The position update formula for followers is as follows:

13 $$ {X_{{i,j}}^{{t + 1}}  = \left\{ {\begin{array}{*{20}l}    {Q \cdot exp\left( {\frac{{X_{{worst}}^{t} }}{{X_{{i,j}}^{t} }}} \right)} & {i > {\raise0.7ex\hbox{$n$} \!\mathord{\left/ {\vphantom {n 2}}\right.\kern-\nulldelimiterspace} \!\lower0.7ex\hbox{$2$}}}  \\    {X_{p}^{{t + 1}}  + \left| {X_{{i,j}}^{{t + 1}}  - X_{p}^{{t + 1}} } \right| \cdot A \cdot L} & {else}  \\   \end{array} } \right.} $$

where $${X}_{P}$$ is the current global optimum, $${X}_{worst}$$ is the present global worst, $$A$$ is a $$1\times d$$ matrix and satisfies that the values in the multiverse are randomly designated 1 or − 1, $${A}^{+}={A}^{T}{\left(A{A}^{T}\right)}^{-1}$$. When $$ i > {\raise0.7ex\hbox{$n$} \!\mathord{\left/ {\vphantom {n 2}}\right.\kern-\nulldelimiterspace} \!\lower0.7ex\hbox{$2$}} $$ , This implies that the individuals with lower fitness values did not receive sufficient food and were left in a state of hunger, thus compelling them to search for additional food resources in other locations.

During the simulation experiment, a subset of sparrows possessed the ability to detect potential threats, known as alert individuals. These sparrows constituted approximately 10 to 20 percentage points of the total sparrow population. The initial positions of the alert individuals were randomly assigned throughout the entire population. The positions of the alert individuals were updated according to the following equation:

14 $$ X_{{i,j}}^{{t + 1}}  = \left\{ {\begin{array}{*{20}l}    {X_{{best}}^{t}  + \beta  \cdot \left| {X_{{i,j}}^{t}  - X_{{best}}^{t} } \right|} & {f_{i}  > f_{g} }  \\    {X_{{i,j}}^{t}  + K \cdot \left[ {{\raise0.7ex\hbox{${X_{{i,j}}^{t}  - X_{{worst}}^{t} }$} \!\mathord{\left/ {\vphantom {{X_{{i,j}}^{t}  - X_{{worst}}^{t} } {\left( {f_{i}  - f_{w} } \right) + \varepsilon }}}\right.\kern-\nulldelimiterspace} \!\lower0.7ex\hbox{${\left( {f_{i}  - f_{w} } \right) + \varepsilon }$}}} \right]} & {f_{i}  = f_{g} }  \\   \end{array} } \right. $$

When ${f}_{i}>{f}_{g}$, the sparrows were located at the boundaries of the entire population and were readily assaulted by external threats. When ${f}_{i}={f}_{g}$, the sparrows in the middle position felt threatened and would try to approach other sparrows as much as possible to minimize their own risk.

Where ${X}_{best}$ is the global optimum, $\beta$ represents the step size control parameter and satisfies the normal distribution, $K\in \left[-\mathrm{1,1}\right]$ is a random number, $\varepsilon$ is the minimum constant value, ${f}_{i},{f}_{g}$ and ${f}_{w}$ are respectively expressed as fit value, the global best fit value and the global worst fit value.

### ISSA

#### Elite opposition based learning (EOBL)

The diversity in the starter population plays an essential role in improving search efficiency, reducing computational time, and enhancing global convergence in the SSA. In order to enhance the diversity of the initial population, the search range of the algorithm is expanded through the implementation of OBL^21^ is introduced to expand the search range of the algorithm. This strategy generates new individual positions based on the positions of existing individuals, thereby enhancing the diversity of the initial population. Introducing backward solutions can expand the search range of the algorithm, but backward learning has certain limitations. It should be noted that the search space where backward solutions exist is not necessarily more advantageous for the current solution. For example, conducting backward search for individuals whose fitness values are higher than those of backward solutions would result in time wastage, thus emphasizing their search in the original domain is more appropriate. Conversely, individuals with fitness values lower than those of backward solutions would benefit more from backward search than from their development in the current domain. To address this issue, we introduce the Elite strategy.

EOBL^22^ utilizes elite individuals to perform backward learning by incorporating their valuable information to generate elite backward solutions, guiding the search process towards the optimal solution. We select exceptional individuals from the current solution and elite backward solutions as the targets for elite opposition-based learning in the next generation population.

Elite backward solution definition: Let ${X}_{i,j}^{E}=\left({X}_{i,1}^{E},{X}_{i,2}^{E},\cdots {X}_{i,d}^{E}\right),\left(i=\mathrm{1,2},\cdots ,N\right),\left(j=\mathrm{1,2},\cdots ,d\right)$ be an elite individual in the d-dimensional search space. The backward solution is defined as $\overline{{X }_{i,j}^{E}}=\left(\overline{{X }_{i,1}^{E},}\overline{{X }_{i,2}^{E}},\cdots ,\overline{{X }_{i,d}^{E}}\right)$; where:

15 $$\begin{array}{l}\overline{{X }_{i,j}^{E}}=c\cdot \left(l{b}_{j}+u{b}_{j}\right)-{X}_{i,j}^{E}\end{array}$$

In the Eq. (15), ${X}_{i,j}^{E}\in \left[l{b}_{j},u{b}_{j}\right],c\in \left[\mathrm{0,1}\right]$ is a random number, $u{b}_{j}=\mathrm{max}({X}_{i,j})$ , $l{b}_{j}=\mathrm{min}({X}_{i,j})$ are determined as the maximum and minimum of the values, For individuals that exceed the search boundaries, the following formula (14) is used for resetting:

16 $$\begin{array}{l}\overline{{X }_{i,j}^{E}}=rand\left({b}_{j}+u{b}_{j}\right)\end{array}$$

#### Golden sine algorithm (golden-SA)

The Golden-SA is an optimization algorithm proposed by Tanyildizi et al.^23^. Its fundamental principle is based on the concept of the sine function, and it is known for its simplicity, ease of operation, and excellent convergence performance. The most remarkable feature of this algorithm lies in its ability to traverse all sine values on the unit circle. By introducing the golden ratio coefficient, individual solutions undergo a significant reduction in the solution space during the iteration process, thereby achieving more comprehensive optimization. This particular characteristic allows the algorithm to converge faster, leading to improved efficiency in the optimization process.

Furthermore, the Golden-SA adeptly balances the trade-off between global exploration and exploitation. It not only extensively explores the solution space to find better solutions but also engages in deeper exploitation during the optimization process, endowing the algorithm with a more comprehensive and robust optimization capability.

By incorporating the Golden-SA mechanism to enhance the explorer's search strategy, the updated approach can be described as follows:

17 $$ \begin{array}{*{20}l} {X_{i,j}^{t + 1} = \left\{ {\begin{array}{*{20}l} {X_{i,j}^{t} \cdot \left| {\sin r_{1} } \right| + r_{2} \cdot \sin \left( {r_{1} } \right)\left| {x_{1} \cdot X_{best}^{t} - x_{2} \cdot X_{i,j}^{t} } \right|} & {\left( {R_{2} < S} \right)} \\ {X_{i,j}^{t} + QL} & {\left( {R_{2} \ge S} \right)} \\ \end{array} } \right.} \\ \end{array} $$

#### Lévy

The Lévy flight, proposed by mathematician Paul Lévy, is a random movement approach characterized by a probability distribution of flight step lengths that follows a heavy-tailed distribution. Incorporating Lévy flight into the enhanced SSA method considerably mitigates the risk of being trapped in local optima, expands the exploration range at the local level, and enhances the algorithm's optimization capabilities. The modified formula is presented below:

18 $$ X_{{i,j}}^{{t + 1}}  = \left\{ {\begin{array}{*{20}l}    {levy\left( d \right) \cdot X_{{best}}^{t}  + \beta  \cdot \left| {X_{{i,j}}^{t}  - levy\left( d \right)X_{{best}}^{t} } \right|} & {f_{i}  > f_{g} }  \\    {X_{{i,j}}^{t}  + K \cdot \left[ {{\raise0.7ex\hbox{${X_{{i,j}}^{t}  - X_{{worst}}^{t} }$} \!\mathord{\left/ {\vphantom {{X_{{i,j}}^{t}  - X_{{worst}}^{t} } {\left( {f_{i}  - f_{w} } \right) + \varepsilon }}}\right.\kern-\nulldelimiterspace} \!\lower0.7ex\hbox{${\left( {f_{i}  - f_{w} } \right) + \varepsilon }$}}} \right]} & {f_{i}  = f_{g} }  \\   \end{array} } \right. $$

where $d$ represents the dimension of the vector. The formula for Levy Flight is calculated as follows.

19 $$ {levy\left( d \right) = 0.01 \cdot \left( {{\raise0.7ex\hbox{${r_{1}  \cdot \sigma }$} \!\mathord{\left/ {\vphantom {{r_{1}  \cdot \sigma } {\left( {\left| {r_{2} } \right|} \right)}}}\right.\kern-\nulldelimiterspace} \!\lower0.7ex\hbox{${\left( {\left| {r_{2} } \right|} \right)}$}}^{{{\raise0.7ex\hbox{$1$} \!\mathord{\left/ {\vphantom {1 \beta }}\right.\kern-\nulldelimiterspace} \!\lower0.7ex\hbox{$\beta $}}}} } \right)} $$

20 $$ {\sigma  = \left\{ {{\raise0.7ex\hbox{${\Gamma \left( {1 + \beta } \right) \cdot {\text{sin}}\left( {\frac{{\pi \beta }}{2}} \right)}$} \!\mathord{\left/ {\vphantom {{\Gamma \left( {1 + \beta } \right) \cdot {\text{sin}}\left( {\frac{{\pi \beta }}{2}} \right)} {\Gamma \left[ {\left( {\frac{{1 + \lambda }}{2}} \right)} \right]}}}\right.\kern-\nulldelimiterspace} \!\lower0.7ex\hbox{${\Gamma \left[ {\left( {\frac{{1 + \lambda }}{2}} \right)} \right]}$}}\beta  \cdot 2^{{\left( {\frac{{\beta  - 1}}{2}} \right)}} } \right\}^{{{\raise0.7ex\hbox{$1$} \!\mathord{\left/ {\vphantom {1 \beta }}\right.\kern-\nulldelimiterspace} \!\lower0.7ex\hbox{$\beta $}}}} } $$

where $\Gamma \left(x\right)$ is the gamma function, ${r}_{1}$ and ${r}_{2}$ are standard normally distributed random numbers, and ${r}_{1}, {r}_{2}\in \left[\mathrm{0,1}\right]$.

**Figure 3:**
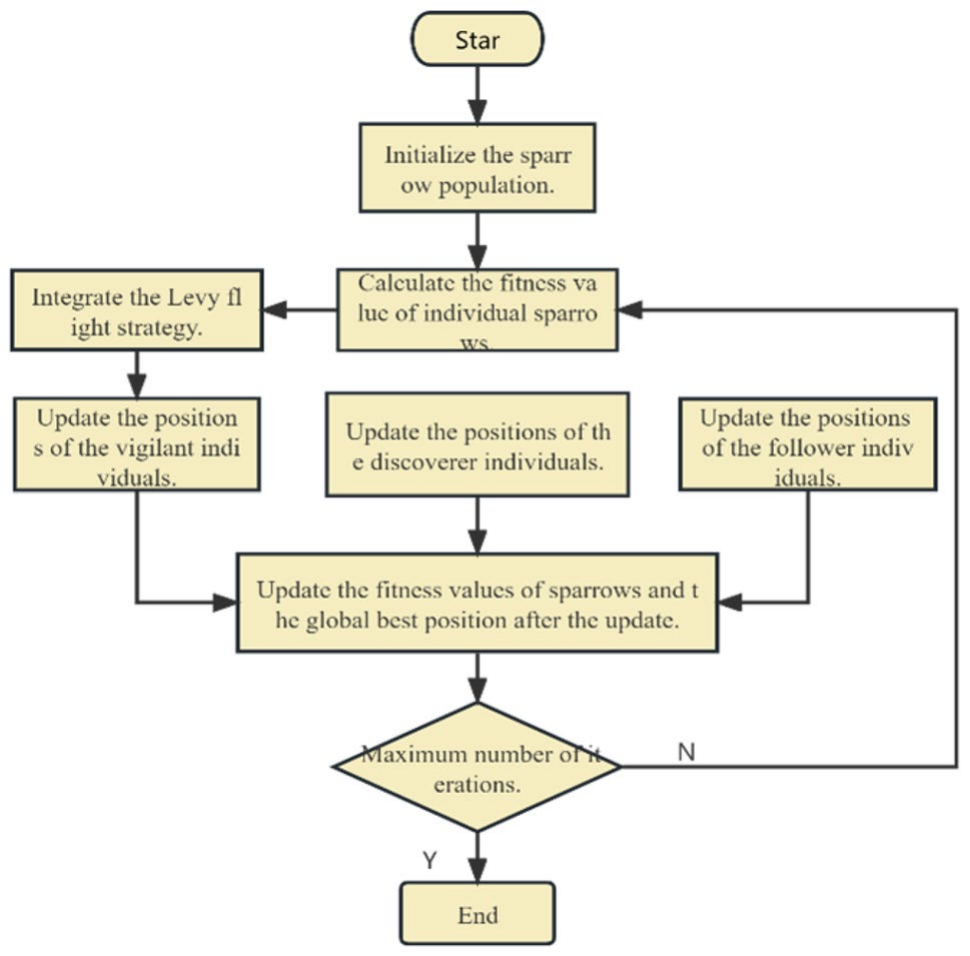
Flowchart of the ISSA.

The SSA suffers from issues of low search diversity and a tendency to get trapped in local optima when solving problems. To address this, one approach to strengthen the performance of the SSA is to incorporate other algorithms. One such improvement method is the utilization of elite backward learning to initialize the sparrow population. This approach leverages prior experiential knowledge to initialize the population, thereby improving the quality of initial solutions. In the enhanced algorithm, called the ISSA, the backward population and the original population are sorted based on their fitness, The top N elite populations were selected to form the next generation of the population. This ensures that the individuals in the population have higher fitness and accelerates the search process. By introducing the Golden Sine mechanism to improve the explorer's search strategy, the model can comprehensively explore high-quality solution spaces. Furthermore, to increase the diversity and comprehensiveness of the search, the Lévy is introduced at the sentinel positions. Lévy flight is a random flight method with long-range jumping capability, and by introducing random jumps in the search space, it helps escape local optima and expands the search range. Figure 3 illustrates the flowchart of the improved ISSA.

**Figure 4:**
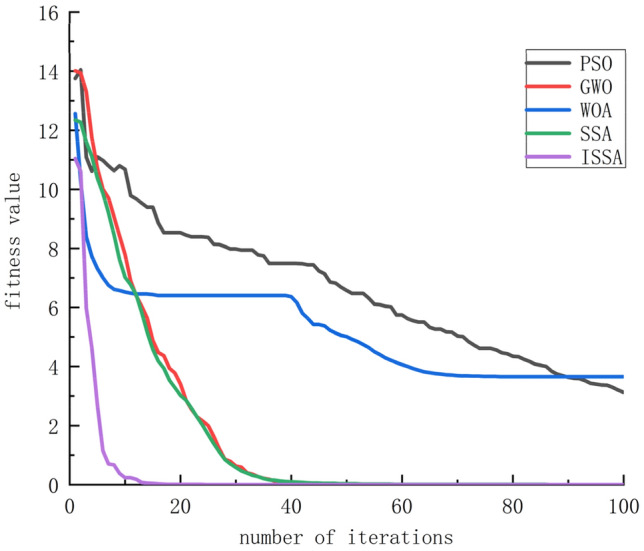
Iteration Comparison of Various Optimization Algorithms.

By experimentally comparing the fitness curves of PSO, GWO, WOA, SSA, and ISSA, the performance of the ISSA algorithm was tested, and the results are shown in Fig. 4^24^. The ISSA algorithm exhibits smaller fitness values, faster convergence speed, and better optimization results, making it more suitable for predicting air quality index.

## Experiments and analysis

### Analysis of data

In this study, two cities, Shenyang and Xi'an, were selected as the experimental subjects from the website tianqihoubao.com. The dataset used for analysis consists of data from January 1, 2021, to December 31, 2021, comprising a total of 365 data points. The dataset includes atmospheric pollutants such as PM_2.5_, PM_10_, SO_2_, NO_2_, O_3_, CO, and NOx, as well as meteorological factors including temperature, relative humidity, wind speed, visibility, maximum temperature, and minimum temperature. The dataset was divided into an 80% training set and a 20% testing set to train the model.

**Figure 5:**
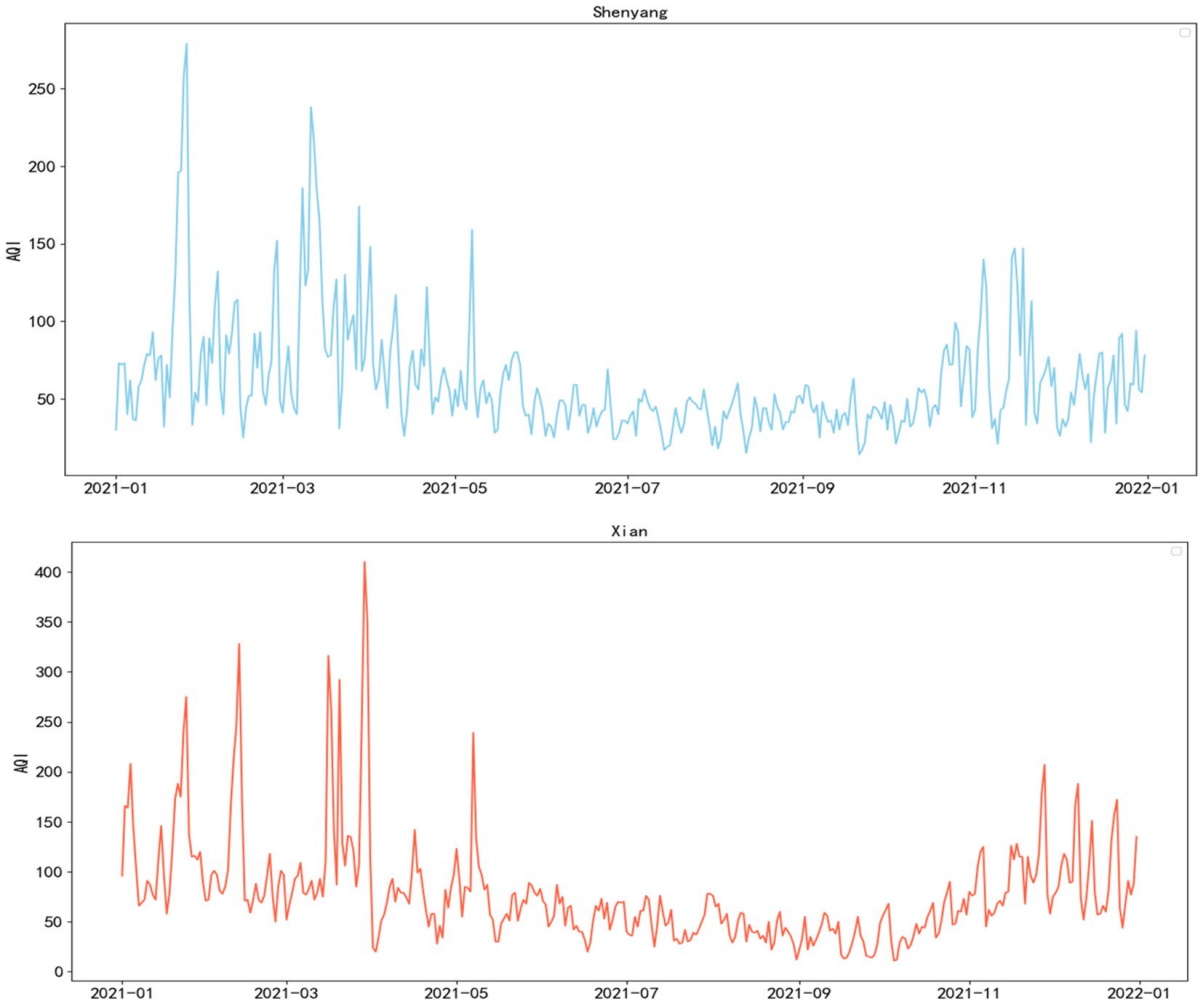
Trends in AQI from 1, 2021 to 12, 2021 in Shenyang and Xian.

To explore the AQI concentration trends in the two cities throughout 2021, the data from both cities were combined to form a continuous time series for a comprehensive trend comparison, as illustrated in Fig. 5.

In the field of machine learning, when dealing with time series prediction tasks, the handling of missing data in the original dataset typically involves two main approaches: data imputation and data removal. Among them, data imputation is the prevailing method, aiming to preserve the temporal continuity of the data and avoid artificially removing data that may impact its temporal nature. This study adopts a combined approach of data imputation and data removal to process the original data and obtain complete pollutant concentrations and meteorological data for the specified research period. Subsequently, data normalization is applied to ensure that all features are uniformly scaled for weighted processing. The normalization formula is as follows:

21 $$\begin{array}{l}{x}^{*}=\frac{{x}{\prime}-min\left(x\right)}{max\left({x}_{m}\right)-min\left({x}_{m}\right)}\end{array}$$

**Figure 6:**
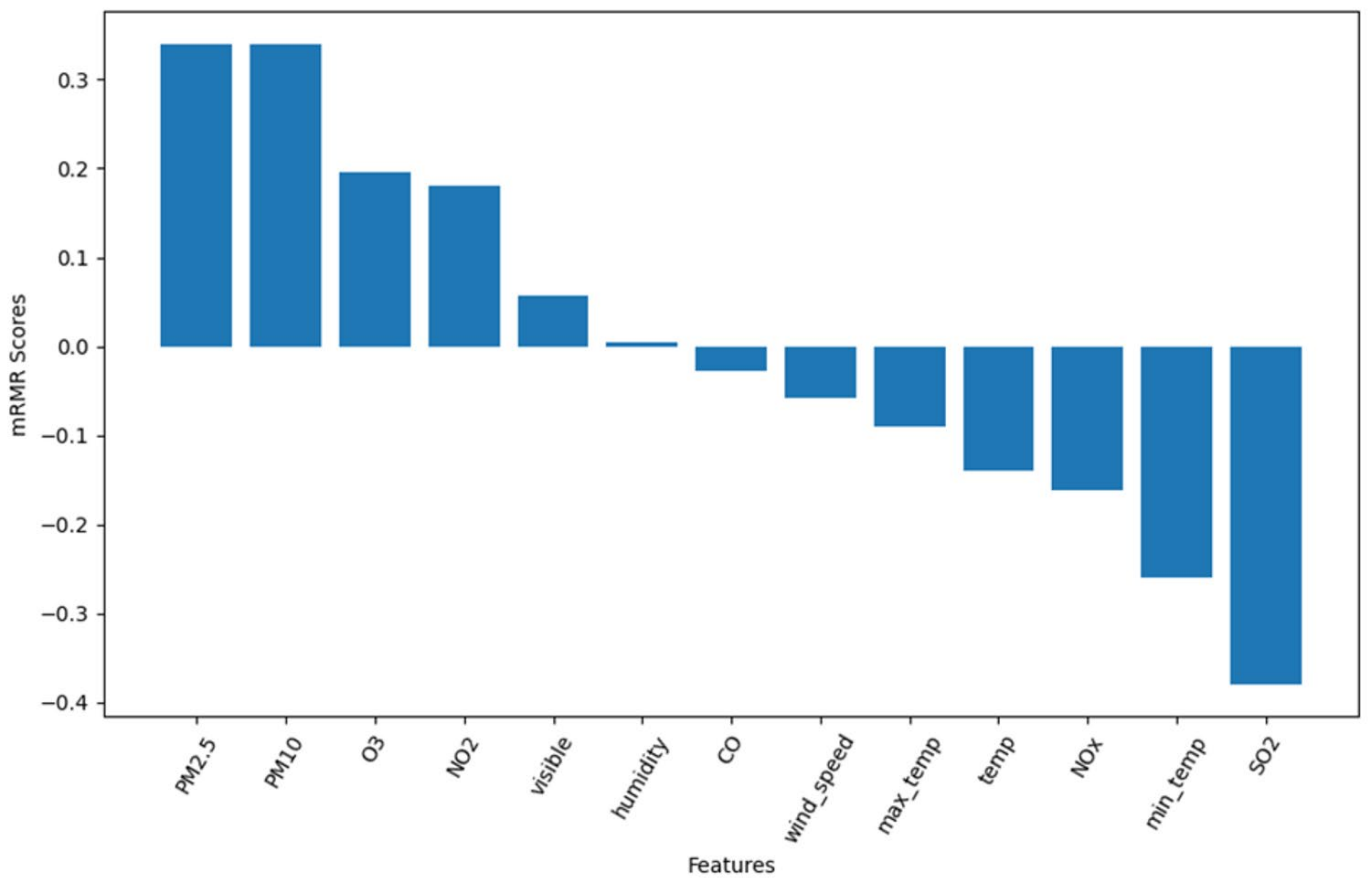
Feature importance ranking based on mRMR.

**Figure 7:**
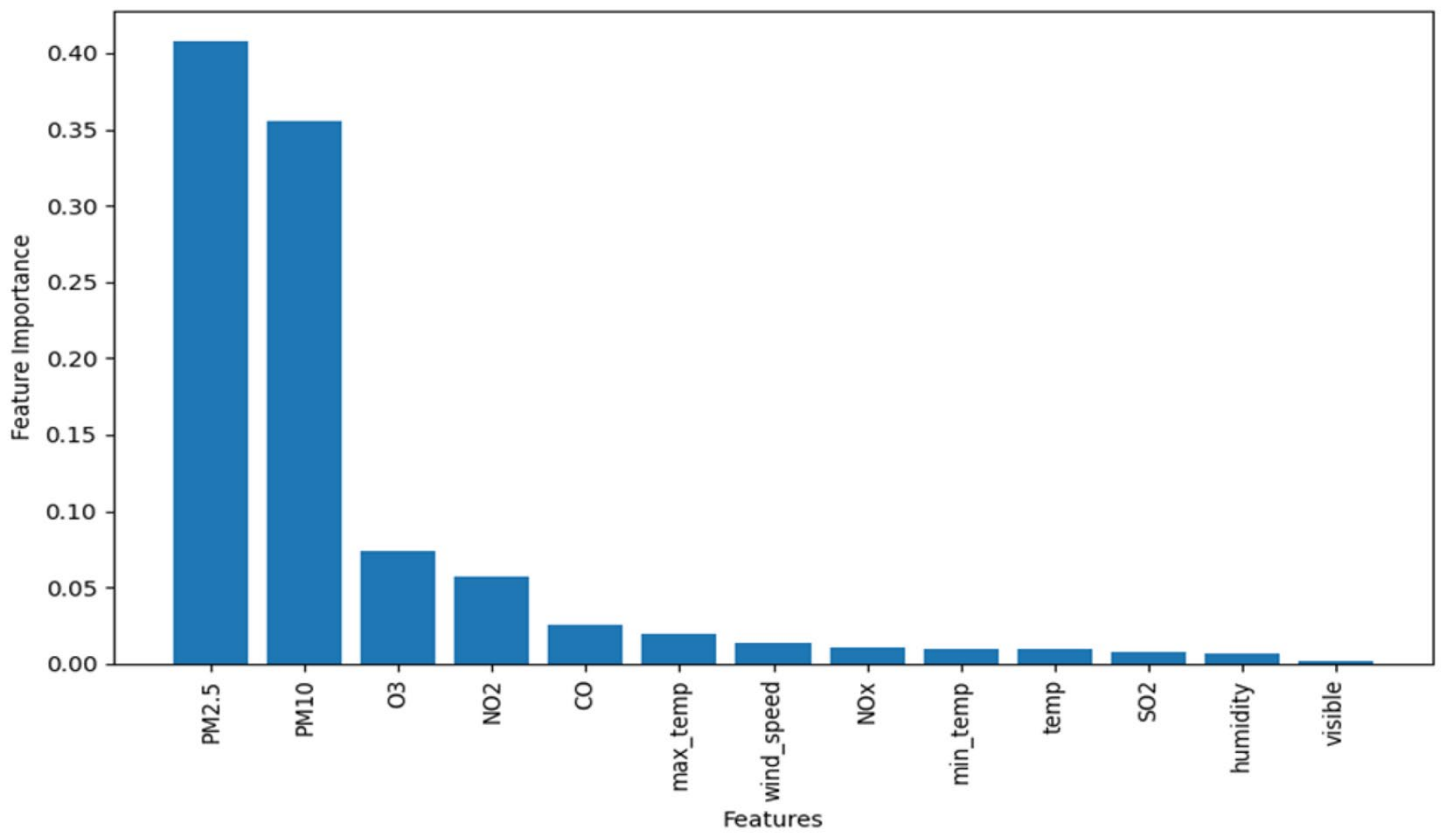
Feature importance ranking based on RF.

When the number of features is too small, it can result in an underfitting model, while an excessive number of features can lead to overfitting and decreased accuracy. Therefore, after data normalization, taking the dataset of Shenyang city as an example, the mRMR algorithm is applied to the processed data for measuring feature relevance and redundancy. This is achieved by calculating the distribution between features and the mutual information between features and the class variable, ultimately obtaining the mRMR scores ranking for each feature. The specific results can be seen in Fig. 6. Subsequently, the RF algorithm is utilized to calculate the out-of-bag (OOB) error values and rank the features, as illustrated in Fig. 7. Finally, the importance scores of features and mutual information scores are combined to obtain a comprehensive score, based on which the features are sorted.

From Figs. 6 and 7, it can be observed that regardless of whether mRMR is used for feature selection or RF is used for importance ranking, the features PM_2.5_, PM_10_, O_3_, and NO_2_ consistently have significant impacts on air quality, ranking among the top four. Taking into account the rankings of each feature from the two algorithms mentioned above, the final feature ranking is obtained and presented in Table 1.

**Table1:** Comprehensive ranking of feature importance.

Feature	Rank
PM_2.5_	1
PM_10_	2
O_3_	3
NO_2_	4
Visible	5
CO	6
Humidity	7
Wind-speed	8
Max-temp	9
Temp	10
NOx	11
Min-temp	12
SO_2_	13

**Figure 8:**
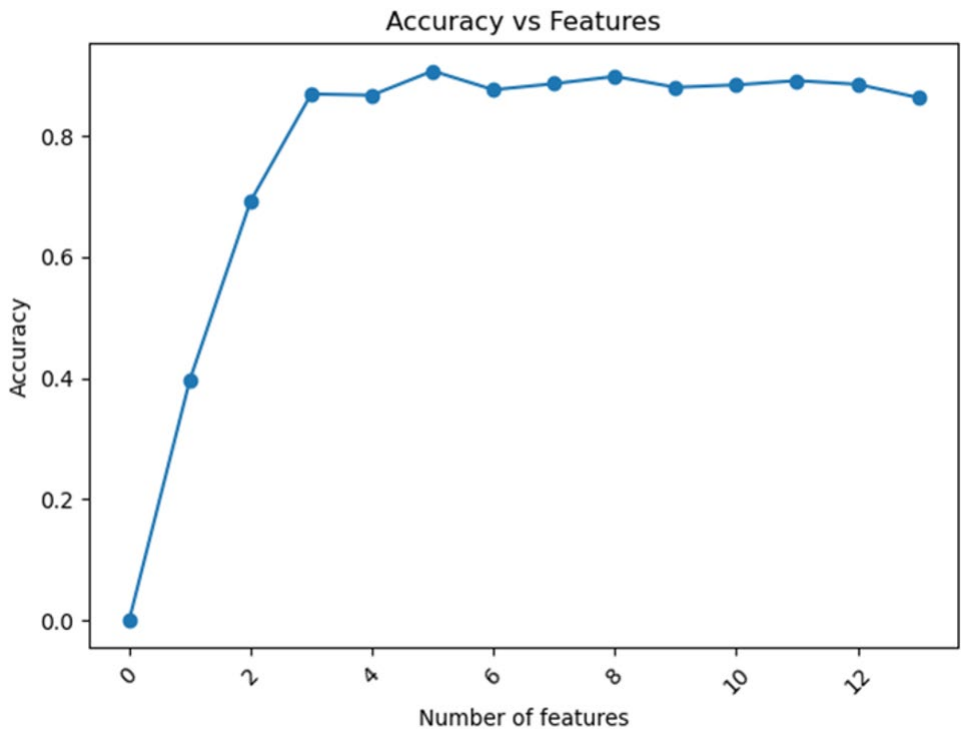
Accuracy values under different characteristics.

In order to find the appropriate number of features, as too few or too many features can affect the model's accuracy, this study conducted experiments with different values of *k*. By comparing the effect of various k values on the model's accuracy, the best *k* value was determined, as shown in Fig. 8. Referring to the outcomes presented in Fig. 8, it can be observed that as the number of features decreases, the model's accuracy tends to increase. This suggests that removing some features with lower importance can reduce the impact of redundant information on algorithm performance, thereby improving the accuracy of predictions. The maximum prediction accuracy was attained with a feature count of 5, further demonstrating the importance of both the quantity and quality of features for model performance. However, as high-importance features are progressively removed, the model's accuracy gradually declines. Therefore, in the feature selection process, it is crucial to strike a balance between reducing redundant information and retaining important features that significantly impact model performance, ensuring the optimal prediction accuracy.

**Figure 9:**
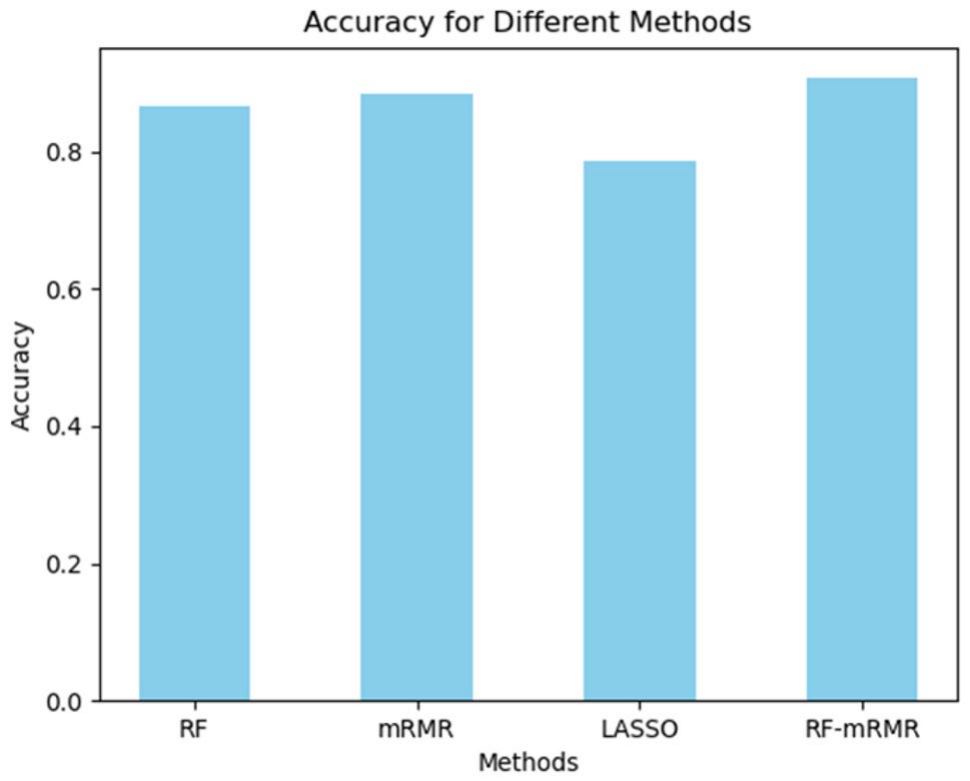
Comparison of experimental results from different feature selection algorithms.

In order to evaluate the efficacy of the feature selection algorithms proposed in this study, a comparison was conducted using the dataset from Shenyang city. The LSTM algorithm was employed for training, The results of the trial are shown in the figure 9. From Fig. 9, it can be observed that the mRMR-RF feature selection algorithm proposed in this study achieved an accuracy of 90.7%, with a selected feature dimension of 5. In terms of accuracy, the proposed feature selection algorithm outperformed RF, mRMR, and LASSO algorithms overall, validating the effectiveness of the feature selection approach proposed in this study.

### Evaluation criteria for experimental results

To evaluate the properties of the air quality index prediction model proposed in this article, this study selected root-mean-square error (RMSE), mean absolute percentage error (MAPE), Mean Absolute Error (MAE), and coefficient of determination (R^2^) as the evaluation criteria for the model's superiority^25^. The equation for computation is as follows:

22 $$\begin{array}{l}RMSE=\sqrt{\frac{1}{n}}  \sum_{i=1}^{n}{\left({\widehat{y}}_{i}-{y}_{i}\right)}^{2}\end{array}$$

23 $$\begin{array}{l}MAPE=\frac{1}{n}\sum_{i=1}^{n}\left|\frac{\stackrel{\wedge }{{y}_{i}}-{y}_{i}}{{y}_{i}}\right|\end{array}$$

24 $$\begin{array}{l}MAE=\frac{1}{n}\sum_{i=1}^{n}\left|{\widehat{y}}_{i}-{y}_{i}\right|\end{array}$$

25 $$\begin{array}{l}{R}^{2}=1-\frac{\sum_{i=1}^{n}{\left({\widehat{y}}_{i}-{y}_{i}\right)}^{2}}{\sum_{i=1}^{n}{\left({y}_{i}-{\overline{y} }_{i}\right)}^{2}}\end{array}$$

In the above four formulas, respectively, $\stackrel{\wedge }{{y}_{i}}$, ${y}_{i}$, and $\overline{y}$ represent the predicted val ues, true values, and mean value of the test set data, where is the sample size of the test set.

### The process of optimizing the hyperparameters of the model

To ensure fairness in the comparative analysis, the ISSA–LSTM model is evaluated and compared to the SSA-LSTM model using the same dataset and testing environment. The proposed model's advantage is verified under identical parameters and experimental conditions. For the random forest, the optimal parameters are determined using the random search method, followed by fine-tuning using the grid search method to further optimize the parameters. The model's accuracy and average error are compared to select the final set of parameters. The chosen parameters for the random forest are presented in Table 2. Regarding the LSTM model, the hyperparameter settings are as follows: the first hidden layer has a range of 1 to 100 neurons, the second hidden layer ranges from 1 to 100 neurons, the iteration count is configured at 100 and the learning rate is set to 0.01.

**Table2:** Parameter settings for random forests.

Parameter	Set point
Number of decision trees	1200
The number of optimal split point features	6
Maximum tree depth	20
The minimum number of samples to delimit	10
The minimum number of samples on the leaf node	2

### Analysis and discussion of results

**Figure 10:**
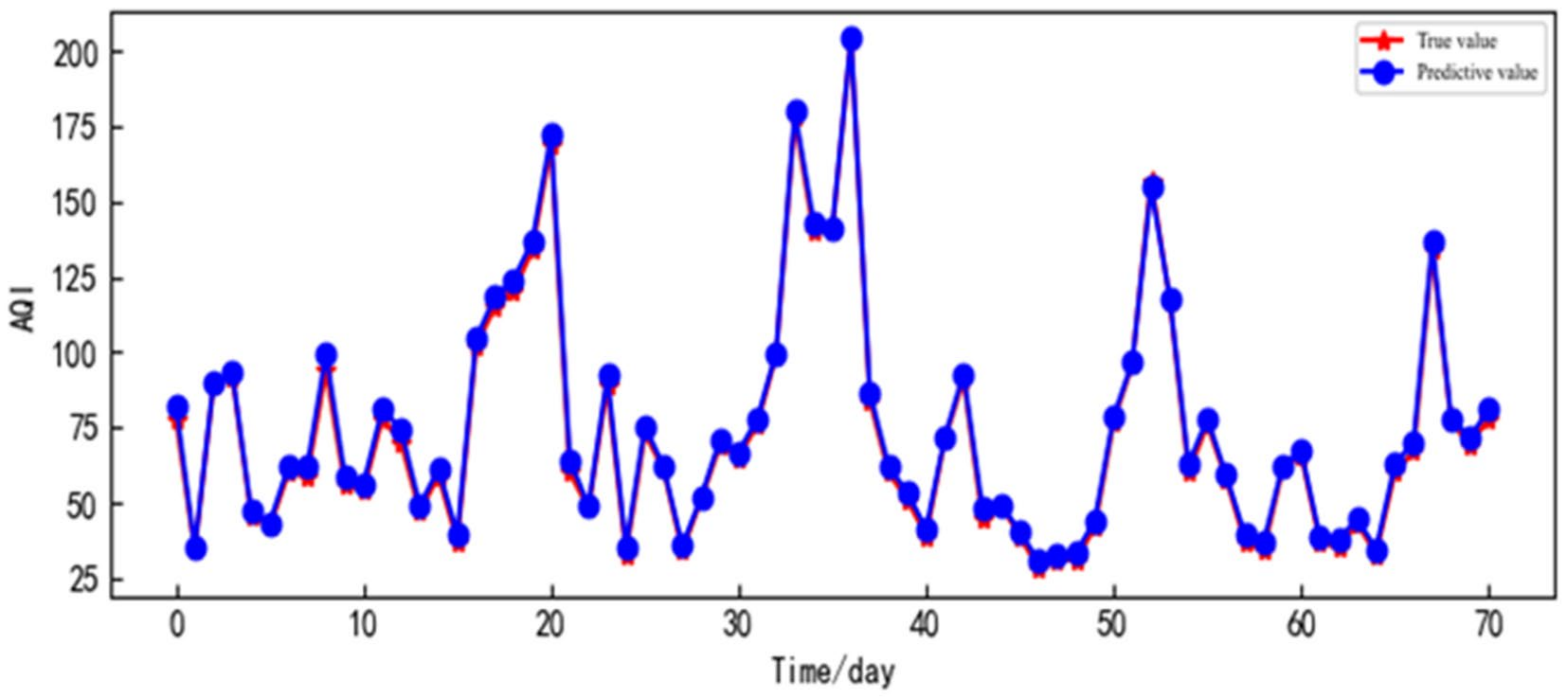
Prediction result of the real value compared with the predicted value of the ISSA–LSTM model in Xi'an City.

The data simulation of Xi'an City was evaluated using the proposed model and the accuracy of the prediction was high. The results are shown in Fig. 10. To showcase the superiority of our proposed prediction model in comparison to other models, we conducted simulation analysis on the AQI data of Xi'an City using various models, including CNN, SVR, BP, LSTM, SSA-LSTM, and ISSA–LSTM. To maintain a fair comparison, all models were trained and tested using the identical dataset. The performance assessment of each model was conducted using metrics like MAPE and R-squared value. The evaluation outcomes for each model are displayed in Table 3.

**Table 3:** Evaluation results of different models for AQI prediction in Xi'an City.

Model	Evaluation index			
	MAPE ($\mathrm{\upmu g}/{\mathrm{m}}^{3}$)	MAE ($\mathrm{\upmu g}/{\mathrm{m}}^{3}$)	RMSE ($\mathrm{\upmu g}/{\mathrm{m}}^{3}$)	R^2^ (%)
SVR	0.221	9.501	11.23	0.891
BP	0.109	6.891	9.815	0.917
CNN	0.064	4.113	5.806	0.971
LSTM	0.103	6.410	8.963	0.931
SSA-LSTM	0.035	2.414	3.144	0.945
ISSA–LSTM	0.013	2.137	2.368	0.996

According to Table 3, it can be observed that the LSTM model exhibits the highest prediction error, with an RMSE and MAPE that are 6.595 and 0.09 higher, respectively, compared to the ISSA–LSTM model. This indicates that hyperparameters have a significant impact on the model. The SVR model performs slightly worse when applied to long time series forecasting, with an RMSE and MAPE that are 8.862 and 0.208 higher, respectively, than the ISSA–LSTM model. The SSA-LSTM model, which utilizes SSA for hyperparameter optimization, results in a reduction of RMSE by 5.819, respectively, compared to the dual-layer LSTM model. However, it still yields higher values of RMSE by 0.776, respectively, compared to the ISSA–LSTM model. The ISSA–LSTM model achieves an AQI prediction accuracy of 99.6%, with an RMSE and MAPE of 2.368 and0.013, respectively, both lower than the other comparative models. This indicates that the ISSA–LSTM model exhibits higher prediction accuracy, thus validating its effectiveness for AQI forecasting.

**Figure 11:**
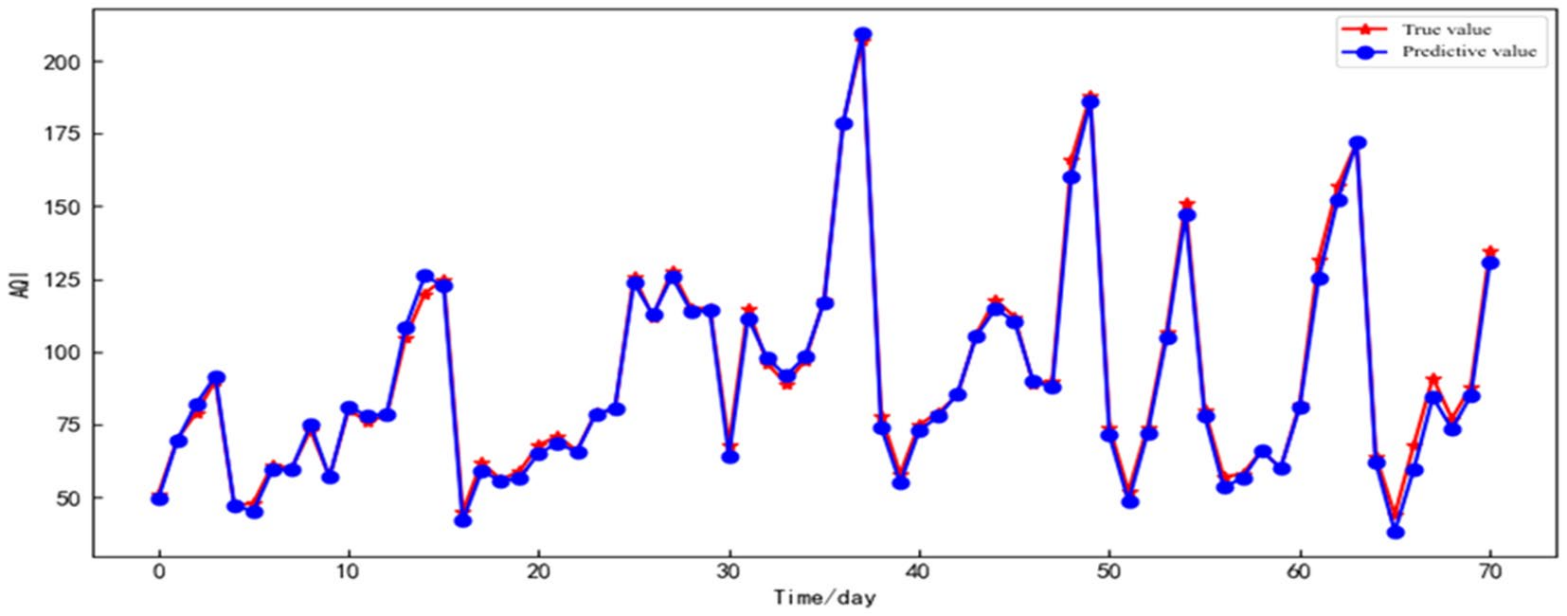
Prediction result of the real value compared with the predicted value of the ISSA–LSTM model in Shenyang City.

Multiple models were utilized for simulation analysis on the AQI data of Shenyang City. The one-step ahead prediction diagram of Shenyang City's AQI using the ISSA–LSTM model is illustrated in Fig.11.The evaluation results of each model are presented in Table 4. The optimized ISSA–LSTM model demonstrated significantly superior performance compared to other models. The improved composite model exhibited even better results, with a decrease in MAPE to 0.028, RMSE reduction to 2.936, and an increased goodness-of-fit with an R^2^ value of 0.996. The accuracy of the ISSA–LSTM model improved by 6.07% compared to the SSA–LSTM model.

**Table 4:** Evaluation results of different models for AQI prediction in Shenyang City.

Model	Evaluation index			
	MAPE ($\mathrm{\upmu g}/{\mathrm{m}}^{3}$)	MAE ($\mathrm{\upmu g}/{\mathrm{m}}^{3}$)	RMSE ($\mathrm{\upmu g}/{\mathrm{m}}^{3}$)	R^2^ (%)
SVR	0.381	21.85	27.96	0.789
BP	0.165	15.36	27.20	0.801
CNN	0.079	6.612	13.18	0.953
LSTM	0.083	7.679	14.95	0.939
SSA-LSTM	0.085	5.071	6.153	0.981
ISSA–LSTM	0.028	2.350	2.936	0.996

**Figure 12:**
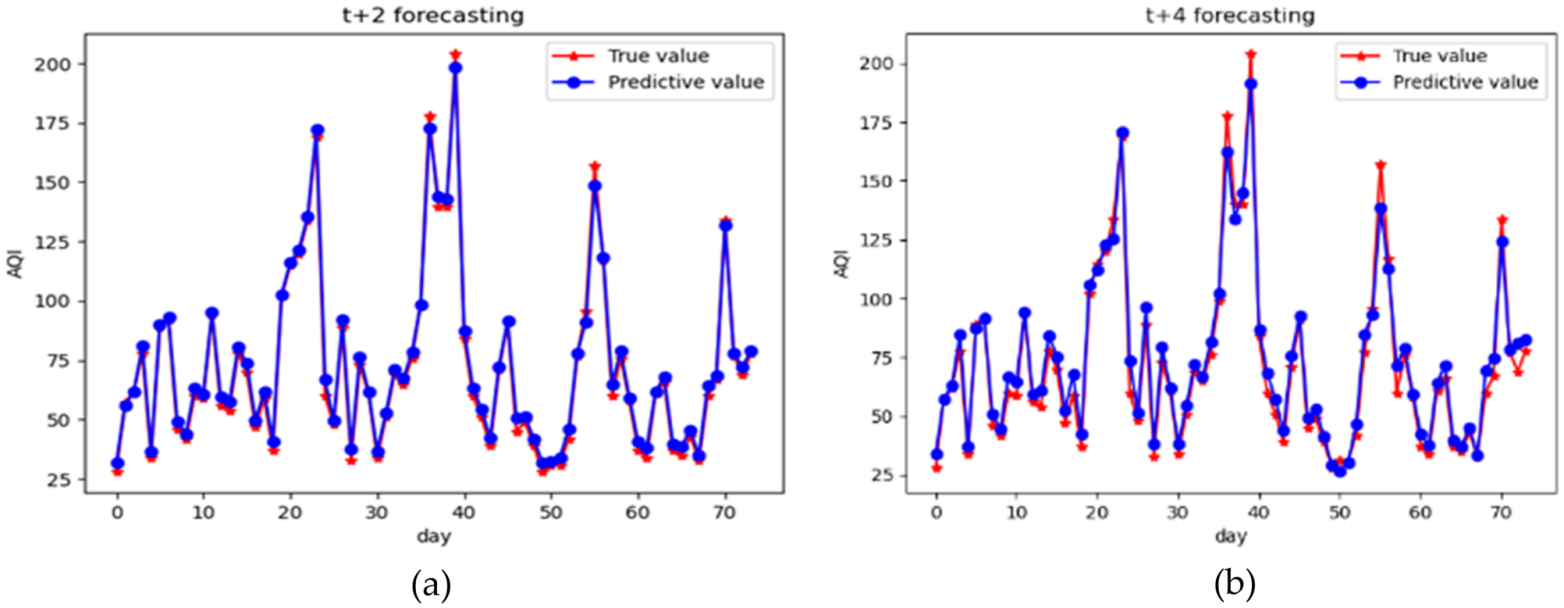
(**a**) is the prediction results for a time step of 2; (**b**) is the prediction results for a time step of 4.

Further investigation of the predictive performance of the ISSA–LSTM model was conducted in this study. The dataset from Shenyang City was selected as an example for air quality prediction with different time steps. Under the same remaining conditions, time steps of 2 and 4 were chosen for air quality prediction. Figure 12 presents the prediction results based on ISSA–LSTM with different time steps, and the error analysis is provided in Table 5.

**Table 5:** Evaluation results of different time step.

Time step	MAPE (µ$\mathrm{g}/{\mathrm{m}}^{3}$)	R^2^ (%)
t	0.028	0.996
t + 2	0.062	0.973
t + 4	0.081	0.925

According to Table 3, it is evident that as the prediction time step increases, the predicted concentrations deviate further from the actual values, resulting in a deteriorating overall prediction performance. The predicted results are significantly inferior to the single-step AQI predictions. The MAPE and R^2^ values for the predictions at 2 and 4 time steps are comparatively worse than the single-step prediction. However, the predictive accuracy of this method can be further improved, for example, by employing more accurate data collection techniques to enhance the precision of the model's predictions. It is believed that with improved accuracy, this prediction model can be applied to various fields.

## Conclusions

In order to further improve the prediction and monitoring of air quality data, it is necessary to conduct in-depth research and exploration of new methods, as accurate short-term air quality prediction is crucial for urban planning and environmental management. Currently, the parameter selection in air quality prediction models mainly relies on empirical knowledge, lacking theoretical foundations and struggling to meet the requirements of accurate prediction. Therefore, in order to enhance prediction accuracy, we introduce the Elite Backward Learning strategy and the Levy Flight-enhanced Sparrow Search Algorithm, combined with the mRMR-RF feature selection method, to optimize the model's key parameters and feature selection process.

Firstly, different air quality data sets are analyzed and processed, and appropriate methods are employed to correct abnormal data. In the process of air quality prediction, we also utilize the mRMR-RF feature selection method, which calculates the relevance between features, to select the most influential features on prediction results, thereby improving the accuracy of the prediction model.

Next, addressing the issue of parameter selection relying on empirical knowledge in existing air quality prediction models, we introduce the EOBL and the Levy Flight-enhanced SSA to optimize the key parameters of the LSTM. The EOBL effectively enhances the global search capability of the algorithm and avoids getting trapped in local optima. Furthermore, the Golden Sine strategy is applied to enhance the explorer's position, thereby improving the algorithm's exploitation capability. The Levy Flight enhancement increases the search diversity of the algorithm, improving convergence speed and accuracy. Through designing simulation experiments based on multiple air quality datasets, the optimization performance of ISSA algorithm was together with other intelligent algorithms. The experimental results indicate that the ISSA algorithm demonstrates faster convergence to superior solutions and attains higher predictive accuracy when compared to conventional algorithms.

Through experimental comparisons, it is evident that the AQI prediction model based on ISSA–LSTM proposed in this study exhibits higher prediction accuracy. Additionally, the prediction performance for AQI at different time steps is also promising.

## Data Availability

The datasets generated during and/or analyzed during the current study are available from the corresponding author on reasonable request.
